# Why not XY? Male monoecious sexual phenotypes challenge the female monoecious paradigm in *Cannabis sativa L.*

**DOI:** 10.3389/fpls.2024.1412079

**Published:** 2024-06-06

**Authors:** Lennard Garcia-de Heer, Jos Mieog, Adam Burn, Tobias Kretzschmar

**Affiliations:** Southern Cross Plant Science, Faculty of Science and Engineering, Southern Cross University, East Lismore, NSW, Australia

**Keywords:** *Cannabis sativa*, sex expression, sex chromosomes, monoecious cultivars, phenotype

## Abstract

Monoecy in *Cannabis sativa* L. has long been considered an industrially important trait due to the increased uniformity it offers and was thought to be exclusively associated with XX females. The isolation and characterisation of a monoecious individual with XY chromosomes sourced from non-proprietary germplasm is reported for the first time. The chromosomal make up of this trait was confirmed through inflorescence structure, growth habit, PCR analysis and sexual phenotypes of progeny from a series of targeted crosses. The identification of an XY monoecious phenotype widens our understanding of monoecy in Cannabis and has important implications for breeding, particularly for producing F1-hybrid seed.

## Introduction

Cannabis is predominantly a dioecious short-day species, meaning that a reduction in photoperiod instigates male and female reproductive phenotypes. However, the species is capable of exhibiting a complex variety of sexual phenotypes, with monoecious individuals and hermaphroditic flowers often observed (Punja and Holmes, 2020). Male inflorescences are cymose panicles with individual flowers hanging from a thin pedicel comprising five tepals and five stamens (Raman et al., 2017). In contrast, female inflorescences develop into a compound raceme of repeating phytomer subunits consisting of two flowers, two bracts, and an axillary shoot (Spitzer-Rimon et al., 2019). A perigonal bract covers each small female flower, hiding the ovary, with the style protruding outwards, ending with two stigmas (Mediavilla et al., 1998). Male and female plants have different developmental timelines in dioecious accessions, with males maturing and senescing earlier (Small, 2017).

The development of modern monoecious hemp (low-THC) varieties largely stems from the introduction of naturally monoecious material from a Central Russian landrace to a Max Plank Institute breeding program in the 1930’s by R. Von Sengbusch (Neuer and von Sengbusch, 1943). These monoecious plants were subsequently inbred and used as the pollen donor to create Fibrimon, and related European fibre type varieties (de Meijer, 1995). Monoecious hemp varieties have traditionally been favoured in industrial contexts and more recently for dual-purpose seed and CBD production due to the increased uniformity and overall crop efficiency (Salentijn et al., 2019). In medicinal crops however, spontaneous monoecious inflorescences are a particular issue, causing detrimental seed formation (Punja and Holmes, 2020). Therefore, the control of monoecious sex expression has long been of interest. The relative expression of female and male flowers, or the degree of monoecy, varies across generations with the potential to revert to dioecy and is quantified with the Sengbusch scale (Faux et al., 2014).

Cannabis is one of the few plant species in which sex chromosomes have been identified (Sakamoto et al., 1998). Cannabis exhibits heteromorphic sex chromosomes, with males being heterogametic XY and females homogametic XX (Moliterni et al., 2004). Karyotyping evidence has shown that the Y chromosome is larger in Cannabis, resulting in a difference in genome size between XX and XY individuals, 1,636 Mbp and 1,683 Mbp, respectively (Sakamoto et al., 1998). Current evidence suggests partial recombination is possible between the X and Y chromosomes in the male parent due to a pseudo autosomal region on the sex chromosomes (Peil et al., 2003).

Chromosomal analysis in two separate studies of a combined 325 monoecious individuals showed an XX chromosomal makeup for all plants, indicating monoecious cannabis plants are genetically female (Faux et al., 2014; Razumova et al., 2016). Supporting the reported chromosomal makeup, monoecious Cannabis plants generally produce the raceme inflorescences typically associated with dioecious females, containing flowers of either sex or a combination of the two, as well as the longer developmental timeline linked with genetic females (Truta et al., 2007).

The current paradigm suggests that monoecy in Cannabis is a dominant X-linked trait, where monoecious individuals are X’X. However, there is currently no direct experimental evidence to validate this assertion (Truta et al., 2007). Research in *Spinacia oleracea* supports this proposition, which also has XX/XY sex chromosomes. Yamamoto et al. (2014) suggest an incomplete dominant gene controls monoecy on the M locus linked to the X/Y loci in spinach. X^m^X and X^m^X^m^ produce monoecious individuals, with the latter having a higher degree of masculinity.

However, both XY and XX Cannabis plants can produce flowers of the opposing sex when specific hormone pathways are manipulated with chemical stimuli, generally known as sex reversion, and the resulting flowers of the opposite sex are fertile (Sriram and Mohan Ram, 1984; DiMatteo et al., 2020). This suggests that both the XY and XX plants have the required genetic architecture to produce flowers of both sexes. Given that manipulation of hormone pathways can instigate sex reversion, monoecy in Cannabis is likely linked to the spatial variability of sex-influencing hormones working upstream to repress or promote floral identity genes (Golenberg and West, 2013). Although this variability thus far has been linked with an XX chromosomal makeup, there is no explanation for why this is or why monoecy should be exclusively an XX-linked trait. Here, we report the isolation and phenotyping of a monoecious XY cannabis plant that challenges the current understanding of monoecy in Cannabis and provides a valuable research tool to understand this economically important trait.

## Results and discussion

**Figure 1:**
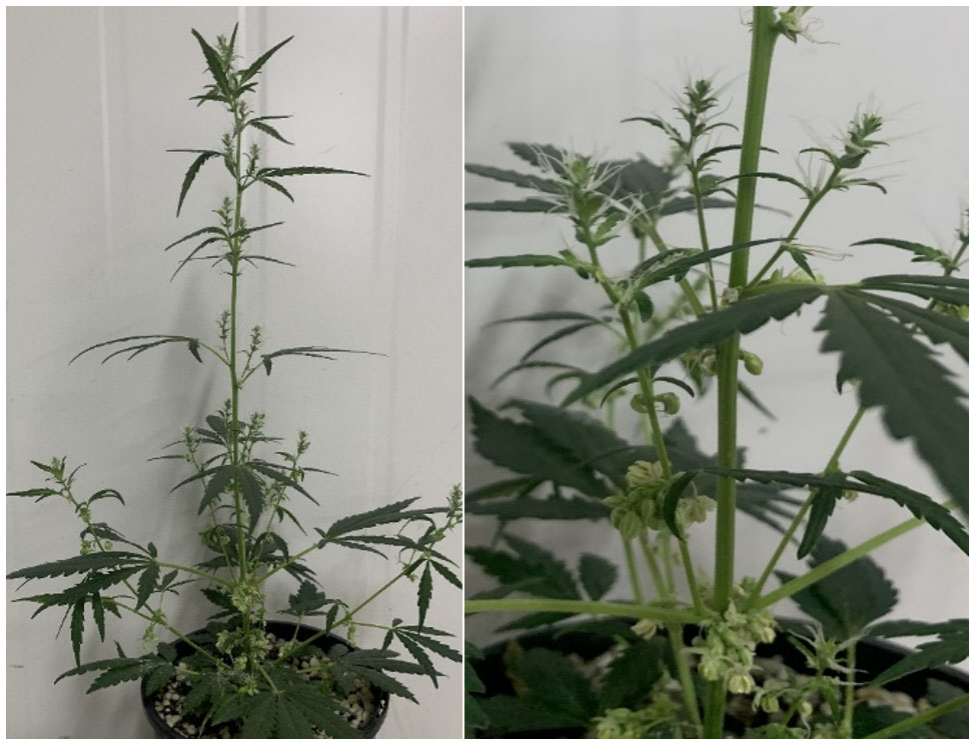
IPK_36 individual exhibiting monoecious sex expression with the male associated panicle inflorescence structure. (Garcia-de Heer, 2022).

The Leibniz Institute of Plant Genetics and Crop Research (IPK) holds a diverse collection of non-proprietary Cannabis accessions. From this collection, an unusual monoecious individual from the IPK_CAN_36 accession was observed (**Figure 1**).

The monoecious individual exhibited a unique flowering structure, with male flowers at lower nodes and female flowers distributed along the upper rachis in an alternate phyllotaxy. Contrary to typical monoecious plants, its inflorescence was a highly branched panicle generally associated with XY males. After isolation and self-pollination, the resulting seeds were germinated and phenotyped, revealing 58 individuals with exclusive female flowers in a compound raceme and 46 monoecious individuals displaying panicle inflorescences, indicating the monoecious trait was fixed through one round of inbreeding, suggesting a simple mode of inheritance These monoecious plants developed male and female flowers across the Sengbusch scale and matured rapidly, desiccating before the seed from dioecious females were ready for harvest, signifying a developmental timeline aligning with male Cannabis. An XY monoecious plant, as the inflorescence structure and maturation rate suggested, would be incongruent with the current understanding of the monoecy phenotype in Cannabis. Alternatively, an XX monoecious plant producing panicle inflorescences would also be unusual. Either case represented an opportunity to gain insight into the genetic mechanisms of monoecy or inflorescence structure in Cannabis and sex determination more broadly.

DNA was extracted from a sub-sample of the progeny and analysed using the male-associated molecular MADC2 PCR markers (Mandolino et al., 1999). Markers resulted in a 390bp amplification associated with a male phenotype in monoecious individuals, while pure females returned results consistent with a female phenotype (**Supplementary Materials S1**). There is some debate on the chromosomal location of the product amplified by the MADC2, original authors postulate it is a non-coding region of an autosome (Mandolino et al., 1999). However, more recent analysis would indicate that these markers do amplify a Y-chromosome specific region (Torres et al., 2022). Regardless, this marker has previously been used on a wide range of genotypes and has successfully differentiated male phenotypes from both dioecious and monoecious females with a very low error rate (Mendel et al., 2016; Razumova et al., 2016; Borin et al., 2021).

From herein, the monoecious individuals producing panicle inflorescences will be referred to as male monoecious (MM). To further investigate the inheritance and chromosomal make-up of the MM trait, a series of crosses were undertaken between IPK_CAN_36 and a stable dioecious accession from the IPK collection, IPK_CAN_57 (**Table 1**). 6mM silver thiosulphate (STS) was applied to Cross 3 to induce male flowers for pollination using a previously published methodology (Lubell and Brand, 2018).

**Table 1:** Crosses that were undertaken to investigate male monoecious (MM) trait and sexual phenotypes of resulting seed (n=100).

	Pollen Donor		Ovule producer		Sexual phenotype of progeny (n=100)			
Cross	Flowering type	XX/XY	Flowering type	XX/XY	Germination rate (%)	Pure Male	Pure Female	MM
1	MM*	XY	Dioecious female*	XX	84	0	35	49
2	MM*	XY	MM*	XY	42	0	24	18
3	Dioecious female*	XX	Dioecious female*	XX	78	0	78	0
4	MM*	XY	Dioecious female**^+^**	XX	68	30	38	0
5	Dioecious male**^+^**	XY	Dioecious female*	XX	60	21	36	3

*indicates individuals from the IPK_CAN_36 accession, ^+^indicates individuals from the stable dioecious IPK_CAN_57 accession.

One hundred seeds of each cross were then grown out, and sex expression phenotyped, with the number of pure males, pure females, and MMs recorded. Crosses 1 and 2 indicate that the MM trait was fixed in the single round of inbreeding from the original plant, with all offspring exhibiting either a pure female or MM sexual phenotype. Cross 4 further confirms that the MM trait is associated with an XY chromosomal makeup, with all progeny being dioecious and 30 individuals exhibiting a sexual phenotype of pure males producing cymose panicle inflorescences. Despite neither parent doing so, cross 5 had some offspring with the MM trait, suggesting that the MM trait is inherited through the mother of the IPK_CAN_36 accession but is only expressed in males.

Combined, the inflorescence structure, PCR results, and sexual phenotypes of the above crosses show that monoecious males exist in Cannabis. This finding has several implications. First, the faster seed development of monoecious males compared to females could prove advantageous in settings with limited favourable growing periods and there is potential to use this trait in contexts where XY monoecious plants may offer yield advantages over traditional XX monoecious plants as either the pollen donors in conjunction with dioecious females for seed production or in dual-purpose fibre, seed crops. Second, MM may allow for easier targetting of important male-linked traits such as fibre quality in potential breeding programs (Horkay and Bócsa, 1996). Third, the shortened developmental timeline of XY males could offer added value in speed breeding, enabling faster cycling of generations compared to their XX counterparts, particularly in developing inbred lines and the downstream applications of F1-hybrid seed production. The MM phenotype would allow for the exploitation of this shortened developmental timeline while reducing the methodological complications of relying on chemical treatments when using a dioecious male to produce inbred lines and regenerating and maintaining the subsequent homozygous seed. This could reduce the cost and increase the scalability of inbreeding programs, aiding the feasibility of producing F1-hybrid seed, which is seen as a critical target in developing Cannabis as a crop (Barcaccia et al., 2020). Fourth, inbreeding XX females leads to exclusively female offspring, whereas inbreeding through an XY individual retains both the X and Y chromosomes, resulting in male and female offspring, allowing for greater flexibility in the end use of the resulting inbred lines. Fifth, the molecular and genetic controls of monoecy in Cannabis is still unclear, and a MM phenotype would offer a unique avenue to further study this trait, potentially contributing to the development of markers able to differentiate between monoecious and dioecious individuals of both sexes. Lastly, this work highlights the potential value of high-quality sequencing of the Y chromosome, which is currently absent in reference genomes, to understand sex determination in Cannabis more comprehensively (Gao et al., 2020).

From an applied perspective, further studies should include testing for linkage drag when introgressing the MM trait and possible negative pleiotropic effects of the MM trait on agronomic performance, particularly in respect to reproductive trait expression.

Given this phenotype is counter to the current understanding relating to the chromosomal makeup of monoecy in Cannabis, and the IPK_CAN_36 is a non-proprietary germplasm, this phenotype presents an opportunity for researchers to gain further insight into the controls and evolution of this industrially important trait.

## Methods

### Plant production

*C. sativa* cultivation, sampling, storage and processing were performed in strict adherence to Sections 23(4)(b) and 41(b) of the NSW Drug Misuse and Trafficking Act 1985, held under the Authority granted to Prof. Bronwyn Barkla of Southern Cross University (SCU), issued by the New South Wales Ministry of Health, Australia. The “IPK genebank collection” was imported under a federal Office of Drug Control (ODC) license to import No. 1820928 and handled under the FAO governed Standard Material Transfer Agreement (sMTA).

The IPK_CAN_36 accession is of an unknown origin, and phylogenetic studies do not group it with other European and Asian accessions from the IPK collection (Woods et al., 2023). Previous metabolite analysis and phenotypic analysis would suggest it is a small-statured, highly branched Type 1 land race (Gloerfelt-Tarp et al., 2023). The first monoecious individual was isolated from a group of 20 plants containing 12 pure males and 7 pure females.

Seed was germinated in hydroponic media (70% coco coir, 30% perlite) supplemented with Osmocote Pro-3-4M (4.3 g/L). Vegetative growth was maintained by growing plants at 26°C under LED lights (300-500 µmol/m^2^/s) with an 18:6 photoperiod. Flowering was induced by introducing plants to a 12:12 photoperiod under HPS lights (1000-1500 µmol/m^2^/s). Where crosses or self-fertilisation were performed, plants were contained in pollen-proof chambers.

### STS treatment

Foliar treatments of 6mM STS were applied at the end of the light cycle on days 1, 3 and 5 of flowering (12:12 photoperiod). Upon application, a branch from each plant was thoroughly coated until dripping (~5ml).

### PCR detection of sex chromosomes

PCR conditions were adapted from Mandolino et al. (1999). PCR was carried out in a 25 μL reaction volume with 5-50ng genomic DNA as template, 0.2 μM Forward and Reverse primers (Sigma-Aldrich) using Platinum Taq (InVitrogen) according to manufacturer’s protocols. The Thermal cycler used was a Veriti 96-Well Thermal Cycler (Applied Biosystems) with the following conditions: 1 cycle at 95°C for 2 mins, 30 cycles of 95°C for 30 secs, 52°C for 30 secs, 72°C for 30 secs and 1 cycle at 72°C for 5 mins. Agarose gel electrophoresis (Bio-Rad equipment) was performed using 1.2% agarose (Benchmark Scientific) supplemented with 5µL/100ml GelRed (Biotium) in 0.5x TBE (Sigma) and run at 100V for 60minutes. The gel image was captured using a GelDoc (Bio-Rad), and PCR product size was estimated using a 1kb DNA ladder (New England Biolabs).

## Data availability statement

The raw data supporting the conclusions of this article will be made available by the authors, without undue reservation.

## Author contributions

LG: Writing – review & editing, Writing – original draft, Methodology, Investigation. JM: Writing – review & editing, Supervision, Resources, Conceptualization. AB: Writing – review & editing, Methodology, Investigation. TK: Writing – review & editing, Supervision, Project administration, Funding acquisition, Conceptualization.
