# Supplementary figures and images for: Why not XY? Male monoecious sexual phenotypes challenge the female monoecious paradigm in Cannabis sativa L
Source: Front Plant Sci. 2024 Jun 6;15:1412079. doi: 10.3389/fpls.2024.1412079 (PMC11187236; doi:10.3389/fpls.2024.1412079)

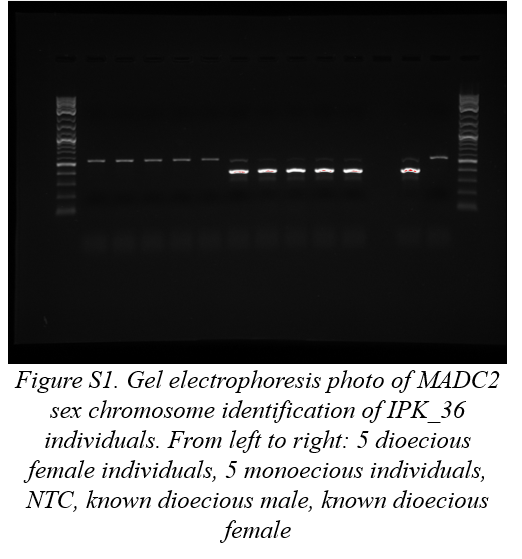

Supplement: Supplementary file 1 [file Image_1.jpeg]
